# Supplementary material for: Control of AMPA receptor activity by the extracellular loops of auxiliary proteins
Source: eLife. 2017 Aug 30;6:e28680. doi: 10.7554/eLife.28680 (PMC5599240; doi:10.7554/eLife.28680)
Supplement: Transparent reporting form [file elife-28680-transrepform.docx]

***eLife’s* transparent reporting form**

We encourage authors to provide detailed information *within their submission* to facilitate the interpretation and replication of experiments. If you have any questions, please contact us: [editorial@elifesciences.org](mailto:editorial@elifesciences.org).

**Sample-size estimation**

- You should state whether an appropriate sample size was computed when the study was being designed
- You should state the statistical method of sample size computation and any required assumptions
- If no explicit power analysis was used, you should describe how you decided what sample (replicate) size (number) to use

Please outline where this information can be found within the submission (e.g., page numbers or figure legends), or explain why this information doesn’t apply to your submission:

We did not compute appropriate sample sizes but followed convention in the field to report at least 5-6 recordings for each condition where practical. Justification for deviation from this norm for patch clamp experiments is described in the methods section (page 25).

**Replicates**

- You should report how often each experiment was performed
- You should include a definition of biological versus technical replication
- The data obtained should be provided and sufficient information should be provided to indicate the number of independent biological and/or technical replicates
- If you encountered any outliers, you should describe how these were handled
- Criteria for exclusion/inclusion of data should be clearly stated
- High-throughput sequence data should be uploaded before submission, with a private link for reviewers provided (these are available from both GEO and ArrayExpress)

Please outline where this information can be found within the submission (e.g., page numbers or figure legends), or explain why this information doesn’t apply to your submission:

The number of replicates is described in the text. Technical and biological replicates are described on line 439. Individual data points are plotted in each graph and reported in source data files. Exclusion criteria for the spot array data are explained in the methods section (page 26). Exclusion criteria for patch clamp data can be found in the Methods section (page 25)

**Statistical reporting**

- Statistical analysis methods should be described and justified
- Raw data should be presented in figures whenever informative to do so (typically when N per group is less than 10)
- For each experiment, you should identify the statistical tests used, exact values of N, definitions of center, methods of multiple test correction, and dispersion and precision measures (e.g., mean, median, SD, SEM, confidence intervals; and, for the major substantive results, a measure of effect size (e.g., Pearson's r, Cohen's d)
- Report exact p-values wherever possible alongside the summary statistics and 95% confidence intervals. These should be reported for all key questions and not only when the p-value is less than 0.05.

The statistical method used is described in the Methods section (page 25). Detailed statistical report for patch clamp experiments is shown in Table 1. Each bar graph presented in Figures 2-7 and relative Supplements shows the values from individual patches. Source data are reported for all summary plots, with exact P-values. We do not currently report effect size, according to the convention in our field. However, work is in progress to make such reporting routine – see github.com/aplested for open source statistics software (DC Stats for Mac) currently in beta testing.

Please outline where this information can be found within the submission (e.g., page numbers or figure legends), or explain why this information doesn’t apply to your submission:

(For large datasets, or papers with a very large number of statistical tests, you may upload a single table file with tests, Ns, etc., with reference to page numbers in the manuscript.)

**Additional data files (“source data”)**

- We encourage you to upload relevant additional data files, such as numerical data that are represented as a graph in a figure, or as a summary table
- Where provided, these should be in the most useful format, and they can be uploaded as “Source data” files linked to a main figure or table
- Include model definition files including the full list of parameters used
- Include code used for data analysis (e.g., R, MatLab)
- Avoid stating that data files are “available upon request”

Please indicate the figures or tables for which source data files have been provided:

We provide source data files for Table 1 (kinetics and steady state currents from electrophysiology) and for Figure 1 we provide the intensities measured from two spot array experiments. Rectification indices for each electrophysiological condition are provided in a separate file for each figure. The six source data files are listed in full on page 28 of the manuscript.
